# A Medical Ethics Framework for Conversational Artificial Intelligence

**DOI:** 10.2196/43068

**Published:** 2023-07-26

**Authors:** Eleonore Fournier-Tombs, Juliette McHardy

**Affiliations:** 1 United Nations University Centre for Policy Research New York, NY United States; 2 London School of Economics London United Kingdom

**Keywords:** chatbot, medicine, ethics, AI ethics, AI policy, conversational agent, COVID-19, risk, medical ethics, privacy, data governance, artificial intelligence

## Abstract

The launch of OpenAI’s GPT-3 model in June 2020 began a new era for conversational chatbots. While there are chatbots that do not use artificial intelligence (AI), conversational chatbots integrate AI language models that allow for back-and-forth conversation between an AI system and a human user. GPT-3, since upgraded to GPT-4, harnesses a natural language processing technique called sentence embedding and allows for conversations with users that are more nuanced and realistic than before. The launch of this model came in the first few months of the COVID-19 pandemic, where increases in health care needs globally combined with social distancing measures made virtual medicine more relevant than ever. GPT-3 and other conversational models have been used for a wide variety of medical purposes, from providing basic COVID-19–related guidelines to personalized medical advice and even prescriptions. The line between medical professionals and conversational chatbots is somewhat blurred, notably in hard-to-reach communities where the chatbot replaced face-to-face health care. Considering these blurred lines and the circumstances accelerating the adoption of conversational chatbots globally, we analyze the use of these tools from an ethical perspective. Notably, we map out the many types of risks in the use of conversational chatbots in medicine to the principles of medical ethics. In doing so, we propose a framework for better understanding the effects of these chatbots on both patients and the medical field more broadly, with the hope of informing safe and appropriate future developments.

## Introduction

With the launch of OpenAI’s GPT-3 model in June 2020 came a new era for conversational chatbots [1]. While there are chatbots that do not use artificial intelligence (AI), conversational chatbots integrate AI language models that allow for back-and-forth conversation between an AI system and a human user. GPT-3, since upgraded to GPT-4, harnesses a natural language processing technique called sentence embedding and allows for conversations with users that are more nuanced and realistic than ever before. The launch of this model came in the first few months of the COVID-19 pandemic, where increases in health care needs globally combined with social distancing measures made virtual medicine more relevant. GPT-3 and other conversational models have been used for a wide variety of medical purposes, from providing basic COVID-19–related guidelines to personalized medical advice and even prescriptions. The line between medical professionals and conversational chatbots is somewhat blurred, notably in hard-to-reach communities where the chatbot replaced face-to-face health care [2].

Considering these blurred lines and the circumstances accelerating the adoption of conversational chatbots globally, we analyze the use of these tools from an ethical perspective. Notably, we map out the many types of risks in the use of conversational chatbots in medicine to the principles of medical ethics. In doing so, we propose a framework for better understanding the effects of these chatbots on both patients and the medical field more broadly, with the hope of informing safe and appropriate future developments.

## The Use of Conversational Chatbots During the COVID-19 Pandemic

During the COVID-19 pandemic, many different types of conversational chatbots were developed. This was both triggered by and an enabler of increased social distancing requirements that also helped support overburdened health systems. This has also allowed public health actors to respond to the “infodemic” of health-related misinformation that has co-occurred with the pandemic with evidence-based health messaging delivered on the same platforms as the misleading or false information [3]. During the pandemic, these chatbots have been useful for disseminating preventive- and vaccine-related messaging, and as tools for triaging, guiding treatment, monitoring symptoms, and providing mental health support for those social distancing or isolating at home [4]. The World Health Organization (WHO) rapidly provided access to its global alerts system via chatbot interfaces on Whatsapp, Facebook, and Viber. It later followed up these efforts by updating its tobacco use cessation virtual assistance, Florence, to provide COVID-19–related advice. The WHO European Regional Office launched, in partnership with UNICEF (United Nations Children’s Fund), HealthBuddy+ to both provide information and allow users to report disinformation and give opinions on the pandemic [5].

Almalki and Azeez [6] had already at the beginning of the pandemic listed nine such uses. A later review found 61 chatbots deployed in response to COVID-19 in 30 countries across areas such as risk assessment, disease surveillance, and information dissemination [7]. Albites-Tapia et al [8] found chatbots being used for the screening and detection of COVID-19 symptoms outside of the health sector including by education providers, retailers, banks, and tourist operators—with 64 cases noted.

## Ethics and Risks in Chatbots for Medicine

Several ethical risks have been documented in conversational chatbots. These include risks related to human rights, such as discrimination, stereotyping, and exclusion; risks related to data, including privacy, data governance, and stigma [9]; and technical risks, such as error tolerance, overconfidence in chatbot advice and decay of trust in health professionals, and, more broadly, technological solutionism [10].

The human rights–related risks are addressed in several recent AI standards. For example, the European Union’s draft AI Act, which was published in April 2021 [11], refers to eight applications of AI that are at higher risk for discrimination: biometric identification; management and operation of critical infrastructure; education and training; employment; access to essential services; policing; migration, asylum, and border management; and administration of justice and democratic processes. It should be noted that AI in health care is not explicitly listed here but is covered elsewhere in the text and in earlier legislation on medical devices [12]. UNESCO (United Nations Educational, Scientific and Cultural Organization) has also developed its Recommendation on the Ethics of Artificial Intelligence, adopted by United Nations member states in December 2021. This document refers to AI in health care and being sensitive to human rights, which member states should monitor quite closely [13]. Going into more depth, the WHO has published guidance on the Ethics & Governance of AI for Health, in which it discusses several risks in medical chatbots, notably in relation to discrimination and privacy [3].

Conversational AI chatbots have several characteristics that could, if improperly used, increase these risks for vulnerable populations. Some of these risks apply to all initiatives collecting data, especially patient data, such as data governance and privacy [14]. Others apply to all AI models, namely, biases in training data, which could lead to the marginalization of certain groups, exclusion of groups in the development and governance of the tool, and error tolerance [15]. Other risks, finally, are unique to the type of AI used, which is natural language processing. Risks in this domain exist in both the interpretation of the input text as well as the construction of the response. Researchers have found many examples of gender and racial stereotyping in GPT-3 and other natural language processing models, which have not yet been corrected by the model owners [16,17]. Some of these risks exist also in other medical applications of AI. However, conversational AI is unique in that it also features specific risks related to large language models [18]. In Table 1, we summarize and illustrate these risks.

**Table 1:** Known risks in conversational chatbots.

Risk category and description			Definition
**Human rights**			
	Discrimination	The chatbot makes different recommendations or has a higher error rate based on the patient’s group (gender, ethnicity, race, religion, etc).	
	Stereotyping	The chatbot interprets or uses language that propagates harmful prejudices, such as the inferiority of certain groups, sexualization, or lack of credibility.	
	Exclusion	Development, governance, or use of the chatbot does not include certain already marginalized groups.	
**Data protection**			
	Lack of privacy	The data generated by the chatbot is not protected.	
	Poor data governance	The data generated by the chatbot is governed improperly or without including the patient.	
	Stigma	The data generated by the chatbot can lead to stereotyping or marginalizing certain individuals.	
**Technical**			
	Error tolerance	Errors, even if they are not discriminatory, cause harm to the patients.	
	Overconfidence and trust decay	Patients place excessive trust in chatbots resulting in overconfidence and relative decay of trust in human health professionals.	
	Technological solutionism	Investment in chatbot technology diverts from an actual societal problem.	

## A Hippocratic Oath for Chatbots

The Hippocratic Oath has undergone many versions and modifications throughout the history of the medical profession. After World War II, a more streamlined version was adopted by the World Medical Association, which was rewritten in 1964 and adopted as the current version in many medical schools globally, although not without some criticism [19]. Broadly, it contains four principles that all health practitioners must adhere to [20]. These principles, therefore, make up the backbone of accepted norms for health professions in many settings and are generally similar to alternative formulations of the leading principles to be applied in medical ethics [19]. Textbox 1 below summarizes these four principles along with their definition.

**Principles of medical ethics.**

**Beneficence**

- Acting for the benefit of patients and promoting their welfare

**Nonmaleficence**

- Not harming the patient

**Autonomy**

- Respecting the patient’s right to and capacity for self-determination (this includes informed consent, truth-telling, and confidentiality)

**Justice**

- Treating patients in a fair and equitable manner

Illustratively, these would play out in conversational artificial intelligence by mitigating the risks such that a chatbot would be able to provide appropriate medical advice without bias or stereotypes, or any of the other risks described.

As we have discussed, the ethical risks of conversational chatbots in medicine have not been mapped out to these principles of medical ethics. However, as we have seen with the recent development of GPT-3, conversational AI is becoming increasingly detailed and realistic, for example, the ability to pass the Turing test [21]. In the deployment of chatbots at scale, in particular during health emergencies, the ethical imperatives of public health focusing on population (rather than individual) health may appear more relevant from the perspective of those designing, commissioning, and delivering them. This is because they will see themselves as institutions delivering often preventative information to large groups of people [22]. From a user point of view, however, these chatbots will often appear to be individual-level interactions and, in certain cases, may substitute partially or entirely for any physician or health practitioner interaction. In analyzing the ethical implications of chatbots, it is necessary to prefer the insider perspective of intended users and the way they will likely construe the interaction [23]. Accordingly, medical ethics may provide a more appropriate framework, which will only become more applicable as AI chatbots grow increasingly realistic and capable of assisting tasks conventionally performed only by health practitioners.

In the section below, we map out the main risks of conversational chatbots for medicine as they relate to the principles of medical ethics. We find that each risk can be related to at least one principle. For example, errors in medical chatbots can lead to harm if they make recommendations, diagnoses, or prescriptions that are wrong. The harm from incorrect diagnoses can then be compounded when chatbots are able to instill such trust in patients that they are unduly confident in the diagnosis, and human health professionals find displacing these erroneous diagnoses in the mind and actions of the patients challenging or impossible [24]. Discrimination can similarly cause harm to certain groups, as well as contravene the principle of justice, since it leads to patients not being treated in a fair and equitable way. Stereotyping similarly leads to direct harm, as discrimination does, and can lead to secondary or societal harms, which might go beyond the medical question (as does stigma). Exclusion is linked to beneficence, in that those that are not represented by the chatbot or cannot use it are not able to access its benefits. Stigma, like stereotyping, can cause harm beyond the immediate medical condition by affecting the patient’s position in society. Lack of privacy and poor data governance can affect the patient’s capacity for self-determination, as well as their right to confidentiality. Overconfidence in technology and trust decay can lead to a lack of adherence to physician guidelines, leading to ill health. Finally, technological solutionism can impact the patient’s ability to receive good care by other means by diverting funds better used to improve in-person health services or address social determinants of health. In Table 2 below, we describe an illustrative relationship between the principles of medical ethics and the risks of conversational chatbots in medicine while also acknowledging that each risk may have a bearing on all of the principles, depending on the implementation.

**Table 2:** Illustration of the framework for chatbot risks to the principles of medical ethics.

Risks	Ethical principles			
	Beneficence	Nonmaleficence	Autonomy	Justice
Errors	—^a^	The chatbot makes the wrong recommendation to patients based on a bug in the system.	—	—
Discrimination	—	The chatbot has a bias that causes it not to understand requests based on women’s health.	—	The chatbot provides more appropriate recommendations for men than for women.
Stereotyping	The chatbot responds to the patient in derogatory terms.	The chatbot’s recommendations based on stereotypes lead to harm the patient.	—	The chatbot gives unfair and derogatory responses to patients.
Exclusion	—	The chatbot excludes certain users because of language and literary skills, withholding medical support.	—	The chatbot excludes certain patients and no alternative is provided.
Stigma	—	Use of the chatbot is not anonymous and leads to stigmatization of certain patients.	—	—
Lack of privacy	—	—	There are data leaks from the chatbot system leading to a breach of confidentiality.	—
Poor data governance	—	—	Patients do not consent to have their data collected by the chatbot and mechanisms for data governance are not clear.	—
Overconfidence and trust decay	—	The chatbot harms the relationship between the patient and their physician by providing contradictory recommendations.	—	—
Technological solutionism	A chatbot is not the best option for providing medical recommendations to certain patients.	—	—	—

^a^Not applicable.

## Applications and Limitations of the Model

This paper provides a simple yet comprehensive framework for the use of conversational chatbots in the health sector. It addresses the extraordinary developments of the last few years in AI conversations and increasing reliance on them due to COVID-19 as well as the likelihood that chatbots will increasingly be used to dialogue directly with people in medical and other health contexts.

In terms of applicability, this framework could be adapted based on locally appropriate norms in medical ethics to underpin an impact assessment process. The use of this process would then be required in assessing and monitoring the deployment of chatbot technology in any circumstance comparable to that of a patient-physician relationship. Concretely, to implement the framework, Table 2 would be used as a guide for practitioners seeking to implement a conversational chatbot, allowing them to reflect on each intersection of risk and principle to consider how this might apply to their tool. This would allow them to consider risks more thoroughly and find solutions to mitigate them before deployment.

As regulatory systems covering AI develop in sophistication to match or exceed what was proposed in the EU Draft AI Act, the results of these medical ethics assessments for chatbots could be required as one component of the reporting requirements for high-risk AI. It is also conceivable that similar medical ethics assessments may be required or beneficial for other deployments of AI in the health sector. However, it should also be emphasized that in other areas, such as when AI is a tool used with a health professional’s mediation, other ethical frameworks such as those of public health or professional responsibility may be more appropriate.

## Conclusion

Over the last few years, conversational chatbot use has increased, driven by a general movement toward the digitalization of health care and public health considerations such as social distancing and remote accessibility. The technology behind conversational chatbots has substantially improved too, first with the Bidirectional Encoder Representations From Transformers (BERT) model developed by Google and more recently with OpenAI’s GPT-3 model, which allows for extremely nuanced and realistic conversations with an AI agent.

At the same time, efforts globally have been made to understand the ethical use of conversational AI, and much research has gone into understanding possible biases, stereotypes, and other uses. Governments globally are in the process of developing regulations that will account for risks in AI technologies to mitigate them. AI regulation, however, does not happen in a vacuum. It will be inspired by existing human rights frameworks, as well as regulations in other domains, such as the European Union’s regulation of medical devices.

The ethical principles of medicine highlighted here in the form of the Hippocratic Oath have informed many regulations around medicine and medical tools globally. It is therefore our hope that this paper will serve to inform the development of a stronger connection between AI ethics and an underlying medical ethics framework, to feed into stronger and more appropriate regulations, and to inform the risk assessment of individual tools.

## Abbreviations

AI: artificial intelligence
BERT: Bidirectional Encoder Representations From Transformers
UNESCO: United Nations Educational, Scientific and Cultural Organization
UNICEF: United Nations Children’s Fund
WHO: World Health Organization
